# Low serum vitamin B_12_ levels among psychiatric patients admitted in Butabika mental hospital in Uganda

**DOI:** 10.1186/1756-0500-7-90

**Published:** 2014-02-17

**Authors:** Michael Ssonko, Henry Ddungu, Seggane Musisi

**Affiliations:** 1 Department of Medicine, Makerere University College of Health Sciences, Kampala, Uganda; 2 Uganda Cancer Institute, Mulago Hospital Complex, Kampala, Uganda; 3 Department of Psychiatry, Makerere University College of Health Sciences, Kampala, Uganda

**Keywords:** Low serum levels, Vitamin B_12_, Psychiatric Illness

## Abstract

**Background:**

Psychiatric manifestations have been noted in patients with low serum vitamin B_12_ levels even in the absence of other neurologic and/or haematologic abnormalities. There is no literature on low serum B_12_ prevalence among Ugandans with psychiatric illnesses. The aim of this study was to establish the prevalence, risk factors, and clinical manifestations of low serum vitamin B_12_ among psychiatric patients admitted in a Mental Health Hospital in Uganda.

**Method:**

Using a cross sectional descriptive study design, 280 in-patients selected by systematic sampling were studied using a standardized protocol. Low serum vitamin B12 was defined as a level < 240 pg /mL.

**Results:**

We found a prevalence of low serum B_12_ in 28.6% of the participants. Absent vibration sense which was significantly associated (58.3% Vs. 26.7%: OR = 3.84 (95% C.I. 1.18, 12.49); p-value = 0.025) with low vitamin B_12_ was observed among 12 participants. Macro-ovalocytes present among 23 participants on peripheral film were significantly associated with low serum levels (73.9% Vs. 26.2%: OR = 7.99 (95% C.I. 3.01, 21.19) p-value < 0.0001). Factors significantly associated with low serum B_12_ levels included psychiatric diagnosis of schizophrenia (AOR 1.74 (95% C.I. 1.00, 3.02); p-value = 0.049), duration of psychiatric illness > or = 3 years (AOR 2.27 (95% C.I. 1.29, 3.98); p-value = 0.004), and hospitalization < 3 weeks (AOR 4.01 (95% C.I. 1.02, 15.79); p-value = 0.047). Female participants were associated with protection from low serum levels (AOR 0.4 (95% C.I. 0.22, 0.73); p-value = 0.003).

**Conclusion:**

Low serum B_12_ is common among hospitalized psychiatric patients with the majority having no haematological findings. Associated risk factors included having a psychiatric diagnosis of schizophrenia, a shorter duration of hospitalization and longer duration of psychiatric illness. Female participants were less likely to have low serum vitamin B_12_ levels. Routine screening for serum vitamin B_12_ levels should be adopted by all hospitals for admitted psychiatric patients.

## Background

Vitamin B_12_ has a very important role in the formation of red blood cells and maintenance of a healthy nervous system. On a complete blood count, a low red blood cell count is often the first sign that points to vitamin B_12_ deficiency. Marked anisocytosis, poikilocytosis, macroovalocytes, basophilic stippling, and hypersegmented neutrophils on a peripheral blood smear are common although not exclusive to vitamin B_12_ deficiency [1].

Psychiatric manifestations can occur in the presence of low serum B_12_ levels but in the absence of the other well-recognized neurologic and haematologic abnormalities [2]. A wide variety of psychiatric disorders which have been associated with deficiency of vitamin B_12_ include dementia, depression, psychosis, schizophrenia, alcohol dependence, mania and obsessive-compulsive disorder [3-11]. On the other hand psychiatric illnesses may predispose patients to vitamin B_12_ deficiencies by changes in appetite, diet, or metabolic requirement [12]. Previous studies have shown that malabsorption due to Giardia lamblia, duration of hospitalisation and psychiatric illness, history of gastro-intestinal surgery, HIV infection, older age and some drugs predispose to low vitamin B_12_ levels [7,8,13-21]. Low serum vitamin B_12_ levels may compromise total recovery of mental health in patients treated for mental illness [22].

Investigators have reported the prevalence of a low serum vitamin B_12_ level among psychiatric inpatients between 5% and 30% [5,8,23]. Total Serum vitamin B_12_ levels are useful in the diagnosis of vitamin B_12_ deficiency [24].

No studies in sub-Saharan Africa have documented the prevalence and correlates of low vitamin B_12_ levels in patients admitted with psychiatric illnesses. The aim of this study was to establish the prevalence, risk factors, and clinical manifestations of low serum vitamin B_12_ among psychiatric patients admitted in a Mental Health Hospital in Uganda.

## Methods

This was a cross sectional study carried out in one centre between January and February 2011 among psychiatric patients admitted at the tertiary referral mental hospital in Uganda. Systematic sampling was used for enrolment of admitted patients from the acute, convalescent, and chronic wards which together had a total number of 650 patients. Probability proportional recruitment according to ward size was used to estimate the number of participants which were recruited from a ward. Using the ward treatment records, a list of patients was generated. The total sample of patients from a ward (**t**) was obtained by multiplying the ratio of number of patients on a ward (**w**) to number of patients in hospital (**T**) with the total sample size number (**N**). Sampling of every **n**^**th**^ patient was selected by **w/t**. The starting participant between 1 and **n**^**th**^ patient was randomly selected. During the five working days in a week, averages of eight patients were recruited per given day. The sample size was calculated through estimation by the Leslie Kish formula with a 95% confidence interval and a precision of 0.05 [25]. The prevalence of low serum levels in hospitalised psychiatric patients was estimated to be 20% [8]. An assumption of 15% was made to cater for eligibility violations and other uncertainties, to make a sample size of 290 participants.

Participants aged 18 years and above who met DSM IV diagnostic criteria for a psychiatric illness were included [26]. Uncooperative patients who declined consent and were aggressive qualified for exclusion from the study.

The outcome variable was level of serum vitamin B_12_ < 240 pg/ml with reference to the kit manufacturer’s recommendations. Independent variables of low serum vitamin B_12_ were known risk factors. Those selected for the study included age, gender**,** duration of psychiatric illness, length of hospitalization and documented HIV serology status [8,14,15,18,19]. Ages considered for participants were their completed years. The difference between the dates which were documented in the patients’ notes when a psychiatric illness was diagnosed and at the time of carrying out the study, produced the duration of psychiatric illness. Length of hospitalisation was estimated from the time the patient had been admitted to the point when the patient was assessed during the study. The patients’ HIV serological statuses were obtained from their in-patient documents. History of medication use and their duration was assessed for drugs like anti-convulsants [20]. Alcohol consumption was estimated using CAGE score with > or = 2 being significant [27]. Others included presence of vitiligo which would point toward an autoimmune disorder, a history of partial or complete gastrectomy and small bowel resection. Psychiatric manifestations were identified according to the up-to-date DSM IV diagnoses made by the attending psychiatrists as was indicated in the in-patients review notes.

Dependent variables entailed the Mini Mental Status Exam (MMSE) scores of < 25, neurologic and haematologic manifestations. Haematologic variables were obtained from the complete blood count and peripheral film report.

### Data collection

We collected data using a pre-tested data collecting tool. Ten patients were interviewed using a designed data tool before commencement of the study. The tool was then adjusted accordingly. A neurologic examination was carried out particularly looking out for vibration sense and joint position sense. The CAGE score and Mini Mental Status Examination for each participant were undertaken using standardised data collecting tools [27,28]. Findings of MMSE scores < 25 were recorded as abnormal and > or = 25 recorded as normal [29,30]. Phlebotomy was done under aseptic conditions.

A blood sample (2-3mls) was collected from each study participant and placed in a plain vacutainer with a clot activator. Samples were stored in a container (cool box) at a temperature of 2-8°C hence their protection from light was ensured. Transportation of samples to the laboratory was done within 24 hours of collection of the sample. Serum samples were assayed using the Cobas E411 according to the manufacturer’s standard operating procedures.

Complete blood count and peripheral film report processing was also done.

### Data analysis

Data was analysed using STATA 10 statistical package. Categorical data was summarized using frequencies whose results were shown using data tables and a bar graph which was used for DSM IV diagnoses. Continuous variables were summarized using means, standard deviations and medians. Univariate analyses using Chi-square tests were performed to determine associations between each independent variable and the outcome variable. Multivariate logistic regression was done to determine the covariates which were independently associated with low serum vitamin B_12_ levels using the Pearson's chi-squared test (*χ*2). A level of significance of covariates with p-values of less than 0.2 at univariate analysis was considered as a qualification for analysis in the multi-variate logistic regression model. A p-value of less than 0.05 was considered significant.

## Ethical approval

The study was approved by the Makerere University College of Health Sciences Research and Ethics Committee as well as Butabika Mental Hospital before any patient was enrolled into the study. Informed consent was obtained from the participants who had insight. For those without insight, their written consent to enrol into the study was obtained from their next of kin.

## Results

**Figure 1:**
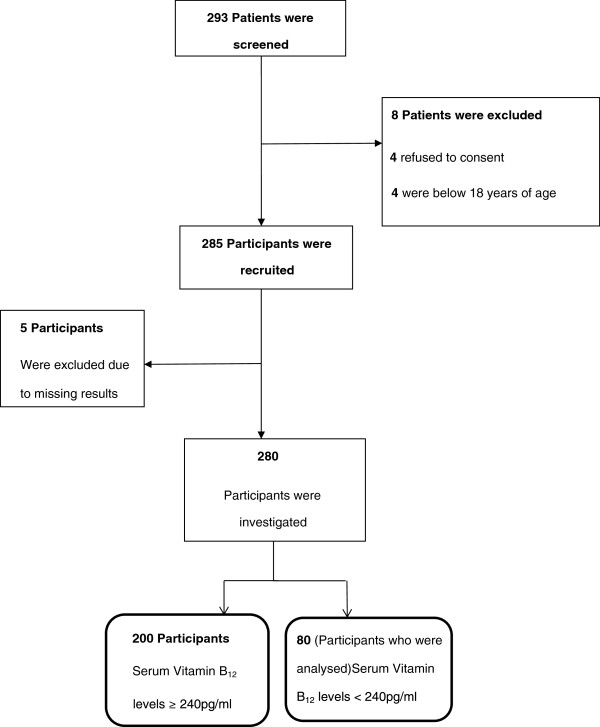
**Screening and recruitment of participants into the Vitamin B12 study.** Of the 13 patients who did not meet the protocol criteria, four did not meet the criteria on the basis of not consenting, and four on the basis of age. Five patients were excluded on the basis of incomplete results.

Between January 2011 to February 2011, 293 patients were approached for consent to participate in the study. Thirteen (4.4%) participants were excluded: four refused to consent, four were below age of 18 years, and five had missing and/or incompatible results (Figure 1). The total number of participants analysed was 280. Among these, 80 (28.6%) had serum vitamin B_12_ levels below 240 pg/ml i.e. low serum B_12_ level. Using the WHO cut off definition (serum B_12_ levels below 203 pg/ml); those with deficiency had a prevalence of 16.4%.

Female participants were associated with protection from low serum vitamin B_12_ levels (17% vs. 35.6%; OR = 0.35 (95% C.I. 0.20, 0.67); p-value = 0.001) compared to the males (Table 1).

**Table 1:** **Serum vitamin B**_**12 **_**levels in relation to the socio-demographic characteristics of study participants**

**Characteristic**	**≥ 240 pg/ml N = 200 (%)**	**< 240 pg/ml N = 80 (%)**	**Odds Ratio (95% C.I)**	**p-value**
Sex				
**Male**	112 (64.4)	62 (35.6)	1	
**Female**	88 (83.0)	18 (17.0)	0.35 (0.20-0.67)	**0.001**
Age				
**≤ 40**	168 (73.0)	62 (27.0)	1	
**41 - 60**	28 (63.6)	16 (36.4)	1.55 (0.79-3.06)	0.207
**≥ 61**	4 (66.7)	2 (33.3)	1.35 (0.24-7.58)	0.73
Residence				
**Urban**	92 (74.2)	32 (25.8)	1	
**Rural**	108 (69.2)	48 (30.8)	1.28 (0.75 - 2.16)	0.362
Type of Residence				
**Own**	151 (69.9)	65 (30.1)	1	
**Rented**	44 (77.2)	13 (22.8)	0.69 (0.35 - 1.36)	0.281
**Institutional**	5 (71.4)	2 (28.6)	0.93 (1.18 - 4.91)	0.931
Employment				
**Yes**	79 (72.5)	30 (27.5)	1	
**No**	121 (70.8)	50 (29.2)	1.09 (1.64 - 1.86)	0.757
Marital Status				
**Married**	48 (73.9)	17 (26.1)	1	
**Cohabiting**	9 (69.2)	4 (30.8)	1.25 (0.34 - 4.61)	0.732
**Single**	124 (70.1)	53 (29.9)	1.21 (0.64 - 2.29)	0.565
**Divorced**	11 (78.6)	3 (21.4)	0.77 (1.19 - 3.10)	0.713
**Widowed**	8 (72.7)	3 (27.3)	1.06 (0.25 - 4.46)	0.938
Religion				
**Muslim**	34 (75.6)	11 (24.5)	1	
**Catholic**	64 (66.0)	33 (34.0)	1.6 (0.72 - 3.54)	0.253
**Anglican**	58 (74.4)	20 (25.6)	1.07 (0.46 - 2.49)	0.883
**Pentecostal**	38 (73.1)	14 (26.9)	1.14 (0.46 - 2.84)	0.781
**SDA**	6 (75.0)	2 (25.0)	1.03 (0.18 - 5.86)	0.973
Level of Education				
**Secondary + above**	76 (71.7)	30 (28.3)	1	
**Primary + below**	124 (71.3)	50 (28.7)	1.02 (0.60 - 1.74)	0.938

C.I = Confidence Interval; SDA = Seventh Day Adventist.

**Figure 2:**
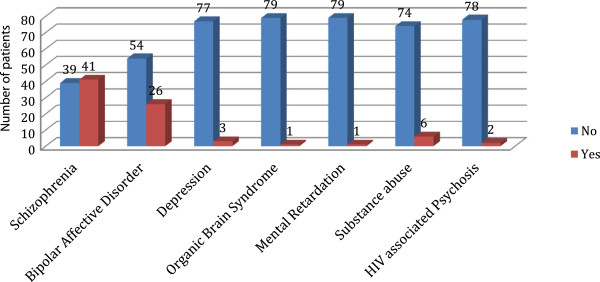
**Distribution of psychiatric illnesses for participants with low serum vitamin B**_**12 **_**levels.** The majority of participants with low serum vitamin B12 had a diagnosis of schizophrenia (41patients). Fifty four participants who had a diagnosis of Bipolar affective disorder did not have low serum vitamin B12 levels.

The distribution of psychiatric illnesses for participants with low serum vitamin B_12_ levels are summarized in Figure 2. The majority of participants with low serum vitamin B_12_ had a diagnosis of Schizophrenia (41patients), followed by Bipolar affective disorder (26 patients).

### Clinical presentations

A total of 12 participants had absent vibration sense which was significantly associated with low B_12_ serum levels (58.3% Vs. 26.7%: OR = 3.84 (95% C.I. 1.18, 12.49); p-value = 0.025).

Mean cell volume of low vitamin B_12_ levels (89.96 ± 9.92 Vs. 85.1 ± 8.5) showed significance (p-value <0.0001). Significant findings in the peripheral film report were hyperchromia (66.7% Vs. 26.7% : OR = 5.48 (95% C.I. 1.80, 16.68); p-value = 0.003), polychromasia ( 66.7% Vs. 26.7%: OR = 5.48 (95% C.I. 1.33, 22.60); p-value = 0.019), macrocytosis (71.4% Vs. 26.6%: OR = 6.90 (95% C.I. 2.09, 22.79); p-value = 0.002), anisocytosis (66.7% Vs. 26.6%: OR = 5.52 (95% C.I. 1.34, 22.73); p-value = 0.018). The majority (23 patients) of participants with significant abnormal findings on peripheral film had macroovalocytes. Subjects with findings of macroovalocytes were associated with low serum B_12_ levels (73.9% Vs. 26.2%: OR = 7.99 (95% C.I. 3.01, 21.19) p-value < 0.0001) as shown in Table 2.

**Table 2:** **Clinical and Laboratory presentations of study participants in relation to serum vitamin B**_**12 **_**levels**

**Variable**	**≥ 240 pg/ml N = 200 (%)**	**<240 pg/ml N = 80 (%)**	**Odds ratio (95% C.I)**	**p-value**
**MMSE**				
Abnormal	118 (71.1)	48 (28.9)	1	
Normal	82 (71.9)	32 (28.1)	0.96 (0.57-1.63)	0.878
**Hyperpigmentation of knuckles**				
No	199 (71.6)	79 (28.4)	1	
Yes	1 (50.0%)	1 (50.0%)	2.52 (0.16-40.77)	0.515
**Joint position sense**				
Present	159 (71.3)	64 (28.7)	1	
Absent	37 (75.5)	12 (24.5)	0.81 (0.40-1.64)	0.553
Not Sure	4 (50.0)	4 (50.0)	2.48 (0.60-10.24)	0.208
**Vibration sense**				
Present	192 (73.3)	70 (26.7)	1	
Absent	5 (41.7)	7 (58.3)	3.84 (1.18-12.49)	**0.025**
Not sure	3 (50.0)	3 (50.0)	2.74 (0.54-13.90)	0.223
**Brisk knee jerks**				
Present	17 (60.7)	11 (39.3)	1	
Absent	183 (72.6)	69 (27.4)	0.58 (0.26-1.31)	0.19
**Absent ankle jerks**				
No	195 (71.7)	77 (28.3)	1	
Yes	5 (62.5)	3 (37.5)	1.52 (0.35-6.51)	0.573
**RBC haemoglobinisation**				
Normochromic	170 (73.3)	62 (26.7)	1	
Hypochromic	22 (91.7)	2 (8.3)	0.25 (0.06-1.09)	0.065
Hyperchromic	5 (33.3)	10 (66.7)	5.48 (1.80-16.68)	**0.003**
Polychromasia	3 (33.3)	6 (66.7)	5.48 (1.33-22.60)	**0.019**
**RBC size**				
Normocytic	171 (73.4)	62 (26.6)	1	
Microcytic	22 (91.7)	2 (8.3)	0.25 (0.06-1.10)	0.066
Macrocytic	4 (28.6)	10 (71.4)	6.90 (2.09-22.79)	**0.002**
Anisocytosis	3 (33.3)	6 (66.7)	5.52 (1.34-22.73)	**0.018**
**RBC shape**				
Normal	172 (73.8)	61 (26.2)	1	
Target cells	20 (90.9)	2 (9.1)	0.28 (0.064-1.24)	0.094
Macroovalocytes	6 (26.1)	17 (73.9)	7.99 (3.01-21.19)	**<0.0001**
**Hypersegmeted neutrophils**				
No	198 (71.5)	79 (28.5)	1	
Yes	2 (66.7)	1 (33.3)	1.25 (0.11-14.01)	0.855
**Haemoglobin g/dL**				
Mean ± SD	12.3 ± 1.79	12.5 ± 1.74	1.07 (0.93-1.25)	0.351
**MCV (FL)**				
Mean ± SD	85.1 ± 8.5	89.96 ± 9.2	1.07 (1.03-1.10)	**<0.0001**

C.I = Confidence Interval; RBC = Red Blood Cell; MCV = Mean Cell Volume; SD = Standard Deviation.

### Known risk factors

Associated risk factors of low serum B_12_ at univariate analysis as shown in Table 3 included three years or more of psychiatric illness (OR 2.34 (95% C.I 1.36, 4.06); p-value = 0.002), and less than three weeks of hospitalization (OR = 3.97 (95% C.I. 1.09, 14.48); p-value = 0.037). Duration of anticonvulsants > 4 years vs. < or = 4 years at univariate analysis was found to be significant (OR = 10.46 (95% C.I. 1.09, 100.59); p = 0.042). A DSMIV diagnosis of Schizophrenia was found significant (OR = 1.8 (95% C.I. 1.08, 3.09); p = 0.024). Forty participants had been prescribed with carbamazepine of which 16 study subjects were found to have low serum levels of vitamin B_12_. Female participants were associated with protection from low serum vitamin B_12_ levels (OR 0.35 (95% CI 0.20, 0.67); p-value = 0.001).

**Table 3:** **Risk factors of study participants and serum vitamin B**_**12 **_**levels**

**Variable**	**<240 pg/ml N = 80 (%)**	**Unadjusted OR (95% C.I.)**	**p-value**	**Adjusted OR (95% C.I.)**	**p-value**
**Sex**					
Male	62 (35.6)	1		1	
Female	18 (17)	0.35 (0.20-0.67)	**0.001**	0.4 (0.22 - 0.73)	**0.003**
**Age**					
≤ 40	62 (27)	1			
41 - 60	16 (36.4)	1.55 (0.79-3.06)	0.207		
≥ 61	2 (33.3)	1.35 (0.24-7.58)	0.73		
**Diarrhoea (> 1 month)**					
No	75 (27.8)	1			
Yes	5 (50.0)	2.6 (0.73-9.24)	0.14		
**Duration of psychiatric illness**					
< 3 years	26 (19.7)	1		1	
≥ 3 years	54 (36.5)	2.34 (1.36-4.06)	**0.002**	2.27 (1.29 - 3.98)	**0.004**
**Duration of hospitalisation**					
≥ 3 weeks	74 (27.4)	1		1	
< 3 weeks	6 (60.0)	3.97 (1.09-14.48)	**0.037**	4.01 (1.02 - 15.79)	**0.047**
**Alcohol consumption**					
No	57 (29.8)	1			
Yes	23 (25.8)	0.82 (0.46-1.44)	0.491		
**Anticonvulsants**					
No	64 (26.8)	1			
Yes	16 (39.0)	1.75 (0.88-3.49)	0.112		
*Duration of anticonvulsants					
≤ 4 years	11 (32.4)	1			
>4 years	5 (83.3)	10.46 (1.09 – 100.59)	**0.042**		
**HIV test**					
Positive	8 (30.8)	1			
Negative	72 (28.3)	1.12 (0.47-2.70)	0.795		
**Schizophrenia**					
No	39 (23.5)	1		1	
yes	41 (36.0)	1.8 (1.08-3.09)	**0.024**	1.74 (1.00-3.02)	**0.049**
**Bipolar affective disorder**					
No	54 (30.5)	1			
Yes	26 (25.2)	0.77 (0.45-1.33)	0.348		
**Depression**					
No	77 (28.9)	1			
Yes	3 (21.4)	0.67 (0.18-2.47)	0.546		
**Organic brain syndrome**					
No	79 (28.7)	1			
Yes	1 (20.0)	0.62 (0.68-5.64)	0.671		
**Mental retardation**					
No	79 (28.4)	1			
Yes	1 (50.0)	2.52 (0.16-40.77)	0.515		
**Substance abuse**					
No	74 (29.4)	1			
Yes	6 (21.4)	0.66 (0.26-1.68)	0.381		
**HIV associated psychosis**					
No	78 (28.9)	1			
Yes	2 (20.0)	0.61 (0.13-2.96)	0.545		

^*^Duration of Anticonvulsants: Participants who took Carbamazepine for the given periods. C.I. = Confidence Interval; OR = Odds Ratio.

Significant correlates at the multivariate logistic regression (Table 3) included Schizophrenia (AOR 1.74 (95% C.I. 1.00, 3.02); p-value = 0.049), participants with a duration of hospitalization < 3 weeks (AOR = 4.01 (95% C.I. 1.02, 15.79); p-value = 0.047), and duration of psychiatric illness > or = 3 years AOR = 2.27 (95% C.I. 1.29, 3.98); p-value = 0.004). Female participants were protected (AOR = 0.4 (95% C.I. 0.22, 0.73); p-value = 0.003) from low serum levels.

## Discussion

In this cross-sectional study, we found a prevalence of low serum vitamin B_12_ levels among admitted psychiatric patients of 28.6% which is high as compared to studies carried out elsewhere [8,31,32]. Currently, there are no agreed upon universal cut off values for the normal range of serum levels of vitamin B_12_[33]. It is possible that the higher prevalence of serum vitamin B_12_ levels may have been aided by the manufacture’s kit recommendations which had reference values based on different population characteristics. The high prevalence of low vitamin B_12_ levels in our study could possibly have been as a result of difference in genetics, patients’ feeding habits or dietary deficiencies which could have been a consequence of the mental illness [8,15]. Use of anti-convulsants is also a possibility [20].

Schizophrenia was independently associated with low serum levels. This could have been aided by the largest population of admitted patients having a diagnosis of schizophrenia. Our findings were similar to other studies [8,23] regarding admitted patients with Schizophrenia. This group of patients is prone to inadequate nutrition during and or before hospitalization which could lead to vitamin deficiencies including vitamin B_12_[7].

Chronic psychiatric illnesses are often associated with repeated lengthy hospitalisations [8,14]. In our study population, factors which could have predisposed participants with psychiatric illness duration of three years or more to low vitamin B_12_ levels included long time use of anti-convulsants, alcohol abuse and frequent and long hospitalizations. These would possibly subject the patients to nutritional deficiencies [15,20]. In Butabika hospital, the diet comprises mainly of cooked maize flour and beans, devoid of beef or poultry feeds. Paradoxically, newly admitted patients of less than three weeks were independently associated with low serum vitamin B_12._ This finding of less than three weeks hospitalization is different to that found in Turkey [14]. It could have been as a consequence of an uncorrected vitamin B_12_ deficiency during or before hospitalization [15]. This also correlates with previous findings of vitamin B_12_ deficiency being associated with acute psychosis [34].

The female population was associated with protection from low serum vitamin B_12_ levels compared to the males. This has been demonstrated in other studies [35-37]. Oestrogen could probably have a protective effect against raised vitamin B_12_ levels [38,39].

The clinical manifestations of participants associated with low serum levels at univariate analysis, included loss of vibration sense. Neurological presentations in our study were best detected by vibration sense which has also been described [40]. Cognitive function as assessed by the minimental status examination did not show evidence of an association with low serum vitamin B_12_. The inconsistent MMSE results among studies may be due to problems of assessment [33].

Haematological findings were abnormal peripheral film report findings of hyperchromic and polychromic RBC haemoglobinisation, macrocytosis and anisocytosis regarding RBC sizes, and macroovalocytes referring to the abnormal RBC shapes. The mean cell volume of 89.96 ± 9.2, a parameter from the complete blood count was also significant. These are the expected haematological findings of B_12_ deficiency [1]. Findings in patients with low vitamin B_12_ levels both on complete blood count (CBC) and peripheral film report were found to be significant in few participants. This alludes to what has been found elsewhere that the initial presentation of low serum vitamin B_12_ levels is a psychiatric presentation before the neurologic and haematological manifestations [12]. This supports the approach of screening for vitamin B_12_ deficiency based on psychiatric presentations and not haematological findings.

Similar to other studies, we found that long term use of anti-convulsants especially carbamazepine (CBZ), led to the development of low serum vitamin B_12_ levels. However in the multi-variate analysis, CBZ was no longer associated with low serum levels. This was due to the few participants (40 patients) who were prescribed the anti-convulsant. This could have contributed to a longer period of four years for an association with low vitamin B_12_ serum levels in this study compared to studies elsewhere [8,20]. Most of the participants, especially those with Bipolar affective disorder used CBZ as their mood stabiliser.

Findings of this study are comparable to research carried out previously [8,14]. Participants’ selection benefited from a wide spectrum of psychiatric illnesses from a national referral mental hospital which is both a primary and secondary unit for the patients.

This research being hospital based, made the study population a selected group of patients whose results may not apply to the general population.

Pernicious anaemia is a known risk factor for low serum levels of vitamin B_12_[41]. For its proper assessment, the anti- intrinsic factor antibody test was not done. Possible autoimmune aetiology was assessed through vitiligo and diarrhoea of more than one month. The sample size for individual diagnostic groups like patients on anti-convulsants was small to allow us make conclusions as previously described [20]. Compliance to anti-convulsants was not assessed. It was not possible to be definitely conclusive as to whether this low vitamin B_12_ levels compromised recovery of these patients.

## Conclusions

Low serum vitamin B_12_ is prevalent among patients admitted with psychiatric illnesses especially those with schizophrenia. Haematologic manifestations typical to low B_12_ serum levels are not as common as expected. Schizophrenia, a long duration of psychiatric illness, and acute hospitalization were independently associated with low serum B_12_ levels among admitted psychiatric patients. The female population was associated with protection from the low serum levels. The associations could be used in the development of a screening tool for selection of preventive strategies. We recommend that patients admitted with psychiatric illnesses should be screened for possible low serum vitamin B_12_ levels.

## Abbreviations

AOR: Adjusted Odds Ratio; CBC: Complete blood count; DSM IV: Diagnostic and Statistical Manual of Mental Disorders, 4th Edition; Hz: Hertz; MCV: Mean Cell/Corpuscular Volume; MMSE: Mini-Mental Status Examination; RBC: Red Blood Cell; SD: Standard Deviation; STATA 10: Statistical Software for Data Analysis Version 10; WHO: World Health Organization.

## Competing interests

The authors declare that they have no competing interests.

## Authors’ contributions

MS contributed to the collection, analysis and interpretation of the data, drafting of the manuscript, critical revision of the manuscript. SM and HD contributed to the interpretation of the data, drafting of the manuscript and critical revision of the manuscript. All authors read and approved the final version of the manuscript.
